# Media reporting of tenofovir trials in Cambodia and Cameroon

**DOI:** 10.1186/1472-698X-5-6

**Published:** 2005-08-24

**Authors:** Edward Mills, Beth Rachlis, Ping Wu, Elaine Wong, Kumanan Wilson, Sonal Singh

**Affiliations:** 1 Dept. of Clinical Epidemiology & Biostatistics, McMaster University, Hamilton, Canada; 2 Centre for International Human Rights Law, University of Oxford, Oxford, UK; 3 Department of Epidemiology, London School of Hygiene and Tropical Medicine, London, UK; 4 Development Studies Institute, London School of Economics, London, UK; 5 Departments of Medicine, Health Policy, Management and Evaluation, University of Toronto, Toronto, Canada; 6 Bloomberg School of Public Health, Johns Hopkins University, Baltimore, USA

## Abstract

**Background:**

Two planned trials of pre-exposure prophylaxis tenofovir in Cambodia and Cameroon to prevent HIV infection in high-risk populations were closed due to activist pressure on host country governments. The international news media contributed substantially as the primary source of knowledge transfer regarding the trials. We aimed to characterize the nature of reporting, specifically focusing on the issues identified by media reports regarding each trial.

**Methods:**

With the aid of an information specialist, we searched 3 electronic media databases, 5 electronic medical databases and extensively searched the Internet. In addition we contacted stakeholder groups. We included media reports addressing the trial closures, the reasons for the trial closures, and who was interviewed. We extracted data using content analysis independently, in duplicate.

**Results:**

We included 24 reports on the Cambodian trial closure and 13 reports on the Cameroon trial closure. One academic news account incorrectly reported that it was an HIV vaccine trial that closed early. The primary reasons cited for the Cambodian trial closure were: a lack of medical insurance for trial related injuries (71%); human rights considerations (71%); study protocol concerns (46%); general suspicions regarding trial location (37%) and inadequate prevention counseling (29%). The primary reasons cited for the Cameroon trial closure were: inadequate access to care for seroconverters (69%); participants not sufficiently informed of risks (69%); inadequate number of staff (46%); participants being exploited (46%) and an unethical study design (38%). Only 3/23 (13%) reports acknowledged interviewing research personnel regarding the Cambodian trial, while 4/13 (30.8%) reports interviewed researchers involved in the Cameroon trial.

**Conclusion:**

Our review indicates that the issues addressed and validity of the media reports of these trials is highly variable. Given the potential impact of the media in formulation of health policy related to HIV, efforts are needed to effectively engage the media during periods of controversy in the HIV/AIDS epidemic.

## Background

With almost 5 million new HIV infections and 3 million AIDS deaths occurring every year worldwide – almost 600 every hour -, the development of safe, effective, and accessible prevention methods has become one of the most urgent global public health needs[1]. One innovative method, aimed at reducing HIV infection through chemoprophylaxis, is the use of the antiretroviral drug tenofovir (Viread), a nucleotide reverse transcriptase inhibitor produced by Gilead Sciences Inc. Tenofovir has been chosen as a promising agent for Pre-exposure Prophylaxis (PREP) therapy for several reasons: it is taken once daily, can be taken without food, and also has a strong safety record, limited side effects, and a favorable resistance profile in HIV patients.

Research in animals indicates that tenofovir used as a PREP may be effective in reducing the risk of HIV infection[2,3], although recent data have questioned the longevity of tenofovir's protective effect if there is one[4]. In order to determine if tenofovir may prevent HIV infection in humans, the National Institutes of Health, the Bill and Melinda Gates Foundation and the Centres for Disease Control funded a series of randomized trials (See Table 1). The conduct of these trials have, however, received widespread criticism by activist groups citing ethical concerns and a lack of community involvement in the planning of the trials[5,6]. This emerging opposition has halted the progress of 2 PREP trials, in Cambodia and Cameroon, and threatened the stability of planned PREP trials, and related recruiting effort, in other developing nations[7].

**Table 1:** Current tenofovir PREP Studies

**Study Location**	**Population Group**	**Sponsor**	**Study Goal**	**Expected Initial Results**
Cambodia	Commercial sex workers 960 volunteers	NIH	Safety & efficacy	2007
Cameroon, Ghana	High-risk women 800 volunteers	FHI	Safety & efficacy	2007
Malawi	High-risk men 500 volunteers	FHI	Safety & efficacy	2007
Botswana	Young adults 1,200 volunteers	CDC	Safety &efficacy	2007
Thailand	Injection drug users 1,600 volunteers	CDC	Safety & efficacy	2007
United States	Men who have sex with men	CDC	Safety	2007
Peru	Men who have sex with men 2,100 volunteers	NIH	Safety & efficacy	2008

### Trial closures

The first trial to close early was a randomized trial to assess the safety and efficacy of tenofovir as a PREP agent in Commercial Sex Workers (CSWs) in Phnom Penh, Cambodia. The trial planned to recruit 960 CSWs and was led by investigators from the United States (US) and Australia. In July 2004, however, a protest staged at the XV International AIDS Society conference in Bangkok, Thailand, brought worldwide media attention to the trials[8,9]. International activist and local representatives of participant groups led the protest. This protest, as well as subsequent demonstrations directed at the Cambodian Ministry of Health, resulted in the early closure of the trial by the Cambodian Prime Minister, prior to recruitment. The Cambodian Ministry of Health has provided no official reasons for their decision to cancel the trial.

In February, 2005, an extension site of the PREP trial in Cameroon was halted by the Cameroon national Ministry of Public Health[6]. The trial was being conducted by Family Health International (FHI) and had begun recruiting participants. At the time of closure, the trial had enrolled 400 high-risk sexual behaviour participants. In this case, media attention again acted as a catalyst to raising concerns about the quality of treatment provided to participants and the quality of care that might be provided afterwards. Protests, led by ACT-UP Paris, an international AIDS activist group based in several countries, and collaborating with Réseau Éthique Droit et Santé (REDS), a Cameroon based AIDS activist group highlighted the concerns with the conduct of the trial and delivery of care[10]. A documentary examining the activists' allegations aired on French TV-2 and made the trial international news. In response to allegations of trial misconduct and ethical violations, the Cameroon government established an independent inquiry into the trial conduct. The independent inquiry reported on February 23, 2005, that the trial cannot proceed without regular reporting and a formal study site accreditation for the satellite trial clinic[11], issues that that had not been addressed by the activist groups or media. The committee did however, identify that many of the allegations made by the media about a lack of safety were false. The inquiry has since recommended that the trials resume after the trial administrators dealt with the reporting issues and attain site accreditation. ACT-UP Paris reports that they will continue to protest the PREP trials taking place in other countries[12].

The media's role as a disseminator of scientific research is particularly important in areas of scientific conduct[13,14]. Because no peer-reviewed publication had been published at the time of conducting this study and the media was a strong catalyst in bringing attention to these trials, we aimed to characterize the nature of their reporting. We determined the sources of information that the media utilized in reporting on the tenofovir trial and the extent to which they consulted activists, researchers and participants involved in the trial.

## Methods

In order to identify all relevant media articles, we searched 3 media databases (POPLINE, PROQUEST and LexisNexis), 5 electronic medical databases (AMED, CINAHL, E-Psyche, EMBASE and MEDLINE) as well as extensively searched the Internet using Google from inception to March 10, 2005. Our specific search terms included, but not limited to: "tenofovir", "viread", "Gilead", "Cambodia" and "Cameroon." We limited articles to those published in the English language. We contacted advocacy agencies and FHI to determine recent interviews they may have provided. We additionally reviewed CDC factsheets.

To be included in our review, articles had to report on the trial controversy and closures in Cambodia or Cameroon. We included articles from any media source, but excluded non-media articles posted on Non-Government Organization (NGO) and activist websites as these contain mostly blogs or position papers and are not intended to be objective. We additionally excluded articles addressing the trial stopped in Nigeria, since this trial was stopped by FHI for logistical reasons, as well as the planned trials of PREP in other developing countries, because they have not been halted at the time we conducted our search [7].

Three authors independently reviewed articles and reviewed them for relevance (PW, BR, EM). Using content analysis, we developed a coding template and extracted the following information from each article: source, date, location of article, author of article, individuals cited, organizations cited, events reported, and source of evidence. We extracted data on the initial coding template through a first reading of the articles to identify major themes. We agreed on the theme categories through consensus. We then reread the articles and coded them appropriately independently, in duplicate. We focused on reporting the claims and counter-claims reported concerning the trials. We aimed to determine the extent to which media reports sought input from stakeholders. As much misleading information was available on the internet through web-blogs, we believed that contact with stakeholders would provide greater inferences into the actual reasons for contentiousness or conduct of the trials. We specifically determined how many articles reported speaking with the following individuals involved in the trial closures: activist groups, researcher groups involved in the trial, local ministry officials and participants. All disagreements were resolved by consensus.

## Results

Our systematic searches yielded 52 relevant articles addressing the trial in Cambodia. We excluded 22, leaving 30 reports, of which 6 are duplicates. In total, we included 24 distinct, original reports addressing the Cambodian trial [15-34]. We included 13 reports that specifically addressed the trials in Cameroon [33,35-45].

### Allegations

#### Cambodia (See Additional file 1)

Thirteen of the Cambodian reports alluded directly to the Prime minister (PM), Hun Sen [5,8,19,21-23,27,29,30,46-48]. The PM was quoted numerous times as being concerned with the study's effects on the human rights of the Cambodian people. With pressure from the Cambodian Sex Workers (CSW) to suspend the study, the PM finally conceded and the trial was halted in August 2004. The representatives from the CSW NGO, Women's Network for Unity, were against using CSWs from poor countries like Cambodia for experimental drug testing. Numerous allegations were made supporting the early termination of the trial and are outlined in Additional file 1. Most were made by CSWs and members of various activist groups opposed to the study. Seventeen reports discussed the demand for 30–40 years of medical insurance to counter any adverse events [15-17,22,25-31,33-35,49,50]. Seventeen papers referred to the claim that some aspects of the study violated basic human rights [5,8,16,19,22-24,27-30,47,48,51]. Twelve reports, in particular, discussed the allegations regarding the participation and potential 'exploitation' of an already vulnerable population [5,16,17,19,23,26,27,29,30,48]. Eleven reports discussed claims made against the study's protocol. For potential participants and many activists, the use of a placebo or a 'dummy pill' predominantly became an ethical concern [5,8,21,24,27,28,30,33,51-53]. Nine reports discussed suspicions regarding the need to run the trial in Cambodia when there were high-risk populations residing in the US and Europe [5,16,19,22,26,30,47,50,52]. Finally, seven reports discussed the allegations that researchers were purposefully providing insufficient counseling and educational resources, ensuring that some women would, in fact become HIV positive during the trial [5,16,23,27,28,33,52]. Of note, one news report published in an academic journal, inaccurately reported that tenofovir was a vaccine[31].

### Counter claims

The demand for long term medical insurance was counterclaimed by an investigator affiliated with the trial in 1 report[50]. She mentioned that this appeal was impossible to meet due to the enormous costs this would entail. She further mentions that there is no place in the world, where participants in clinical trials receive coverage for life. A representative from FHI directly counters the allegations made concerning the violation of human rights[47]. The FHI representative countered that the sex workers involved in the trial are offered an enhanced standard of care well beyond what is typically offered in Cambodia and other HIV prevention trials. No investigators or officials were reported responding to the allegations regarding the use of a placebo nor the claim that counseling is purposely limited to ensure some seroconversions.

#### Cameroon (see Additional file 2)

There were 13 reports addressing the tenofovir trial in Cameroon [12,33,35-37,39-45,54]. Nine reported allegations that there was no care for those who seroconvert during the trial [12,33,35-37,39-41,54]. The charge that participants are not being sufficiently informed of the risks involved was reported in 9 articles [12,33,35-37,40,41,43,44]. Other major allegations included an inadequate number of support staff (6/13) [12,36,39-41,54], participants being exploited and treated like 'guinea pigs'(6/13) [12,36,39-41,54], an unethical study design (5/13) [12,36,40-42,44] and finally, the belief that trial is only being conducted in Cameroon to promote Gilead's commercial prospect (5/13) [12,40-42,44].

### Counter claims

Two reports interviewed investigators who directly responded to the claim that no help is provided for those who seroconvert during the trial [36,43]. The study co-ordinator in Douala was cited, saying all volunteers who have become infected are referred to approved medical centers for treatment[36]. A representatives from FHI in another report [43], responded citing that study-support services are offered and that women can decide if they want to continue on this path. In addition, it was reported that women have access to medical care and treatment including referral to services where they can receive care for HIV. The representative emphasized that women who have volunteered to participate will have "life-long access to HIV care and treatment". To counter the claim that participants are not fully nor sufficiently informed of the risks involved, an FHI representative in one report directly responds to this allegation. She states that all potential participants "were counseled before the trial started to make sure they understood the potential risks and benefits of study participation" [43].

### Sources of information (see Table 2)

**Table 2:** Sources of information cited in media reports

**Study**	**Total**	**Investigator**	**Potential participant**	**Representatives of participant groups**	**Activist group**	**Others**
Cambodia	24	3 (12.5%)	1 (4.1%)	1 (4.1%)	3 (12.5%)	3 (12.5%) NIAID representative; Cambodian AIDS authority; Bio-consultant
Cameroon	13	4 (30.8%)	0	1 (7.7%)	6 (46.2%)	1 (7.7%) Local physician

#### Cambodia

In order to evaluate the soundness of the reports, we identified the source of the information to determine whom, if anyone had been interviewed. Potential subjects for the interviews included trial investigators, potential participants (ie. CSWs), representatives or activist group members. Of 24 reports, 14 did not identify any primary source of information [8,16,21-23,27-30,33,50,52,55,56]. In 3 reports trial investigators or officials were interviewed [47,48,50] All investigators emphasized that the concurrent trial care provided was beyond the standard of care in Cambodia and in-addition to what is offered in other HIV prevention trials.

One report interviewed potential participants [24]. In three reports that cited activists, their comments came from web blogs [5,26,53]. Representatives of NGOs were interviewed in one report [48].

#### Cameroon

Of the 13 reports, 4 did not report a primary source of information[33,35,44,45]. Two reports interviewed both researchers and opponents[36,37]. Four reports concerning the Cameroon trials surveyed investigators[36,37,41,43]. There were no interviews with potential participants in any reports. An interview with a participant representative was recorded in one report [36] and HIV activists in 6 reports, either in person or through Internet forums [12,36,37,40,42,54].

## Discussion

The findings of this review should be of interest to clinical trialists, advocates and policy makers. The media are important communicators of information and can raise awareness of emerging issues concerning health and risk[57] We observed that the media brought forth a variety of concerns related to the conduct of the trials in both Cambodia and Cameroon. In several instances these concerns were not supported by evidence and were possibly inaccurate. We also observed that the media involved stakeholders to a comparatively small degree, suggesting that the viewpoints they reported may not accurately represent the views of all those concerned about the conduct of the trial. Given the potential impact of the media reporting on the conduct of future tenofovir trials, the nature of their reporting deserves closer scrutiny.

There are several limitations to consider in interpreting our review. Although we systematically searched many databases and extensively searched the Internet, it is possible that we were unable to identify some reports. Our searches were limited to the English and French languages, and as both Cambodia and Cameroon have media in other languages, we may have missed articles published in their respective languages. We conducted a content analysis to identify issues that we deemed trial related. It is possible that other issues exist, and that other individuals were interviewed, but were not reported in the articles. There are also several strengths to consider in interpreting our review. This is, we believe, the first systematic review to assess media reporting related to the closed trials. We conducted extensive searches and extracted data independently, in duplicate, to remove investigator driven biases. We spoke with representatives from each stakeholder group to determine if they were aware of additional reports, as well as searched the CDC reports.

The media reports consistently reported concerns with ethical issues in the conduct of the trials. Both the Cambodian reports and the Cameroon reports cite access to appropriate standards of care for those who become infected during the trial and appropriate standard of prophylaxis. Indeed these same issues are being echoed in the impending trial of tenofovir in drug users in Thailand[9]. Further to this, most media reports regarding both trials reported that participants were not fully aware of the risks involved with participation. Clinical trialist's planning prevention trials, whether of chemoprophylaxis or microbicides should be aware that many populations are not in situations in which to make informed decisions about clinical trial methodologies and the risks related to trial participation. Enrolling these communities in developing nations requires the establishment of community advisory boards and gender advisory boards, to ensure that the information is being provided, and interpreted, in an accurate manner[58,59].

The tenofovir trial media attention does however, have implications for prevention trials, such as HIV vaccines. Prevention methods are urgently needed to stem the onslaught of new HIV infections. Novel interventions, such as vaccines, microbicides, or chemoprophylaxis, will all require clinical trial validation. The media reporting of the tenofovir trials may threaten trial recruitment and potentially stigmatize trial participation.

There are important qualitative differences between the media reports of the trial in Cambodia compared to the trial in Cameroon. It is important to note that the trial in Cambodia was closed before recruiting any participants, whereas the trial in Cameroon had met its recruitment goals (n = 400). Most criticisms regarding the trial in Cambodia related to a lack of access to adequate care during the trials and post-trial, for those who may become infected. These concerns for potential infections appeared to be the overriding concerns as activists cited that these trials would be conducted to a different level of protection and insurance if they were conducted in a developed country. The articles addressing the trial closure in Cameroon were somewhat different and were largely based on reports of trial misconduct, in the context of inadequate informed consent, inadequate access to counseling and inadequate access to male and female condoms. The trials did however; have overlapping concerns about the ethical standards for trials in developing nations compared to Western nations and the protection of the participants human rights.

The tenofovir trials raise important questions about the relationship between researchers, participants and activists[60]. With worldwide media attention, these trial closures demonstrate the ability of activists to engage the media and bring about important consequences for the conduct of trials. Activists are experienced at bringing about change and have strong lobbying potential that can impact researchers and the HIV community. The activist communities have substantial experience using the media to address topics of importance in HIV and are well-educated regarding ethical standards and trial designs. Indeed, it is important to note that several of the ethical issues raised in the media reports: standard of care, access to proven prophylaxis and access to treatment for seroconverters; have not yet been resolved within the academic community [61,62].

### The Media, accuracy of communication and influence on policy

The media plays an important role at the interface of science and policy. Indeed, the media has been utilized to change behaviour in HIV public health campaigns[60]. Most scientists and non-scientists receive information from media sources [63]. One policy making model specifically addresses this issue and describes how information is generated by researchers and then disseminated by advocacy networks and the media [64]. The information provided by the media is eventually interpreted by policy makers and government officials, and those informally involved, such as patient groups and stakeholder groups. Although media information provides weak levels of evidence, multiple sources of media information may strengthen personal inferences. The interpretation of this information is influenced by the value systems of the individuals receiving the information.

This review displays the important role that the media play in knowledge dissemination, and the responsibilities that they have in accurate reporting. In our review, 20 reports did not acknowledge interviewing any stakeholder and in no case did any report interview those supporting the trials and those against the trials. It is impossible to determine the extent to which the allegations made regarding the trials were wholly inaccurate. Our review contributes to a large body of evidence indicating that the media at times misrepresents scientific information and presents the science in a varied manner [65], as in the case of the trial in Cameroon where the reports suggest inadequate counseling and informed consent sheets that were not available in the local languages, issues that were not upheld by the ministry of health. It should be noted however, that not only standard news sources, but also academic news sources were susceptible to inaccuracies in their reporting. In one case, a top academic journal reported tenofovir to be a vaccine trial[31].

Indeed the media plays an important role in highlighting HIV issues so that it remains of topic of high profile and can be used as a strategy to convey AIDS awareness and prevention[60]. The media has a responsibility to report in a factual and objective manner [66]. Media agencies should be engaged to educate them on trial protocols and ethics so that reporting can be discriminating. The International AIDS Vaccine Initiative (IAVI) has now initiated training with journalists to ensure knowledgeable reporting related to vaccines [67]. This seems like a logical step towards ensuring adequate reporting of prevention trials.

We recommend further analysis of this emerging issue of media reporting of contentious clinical trials. The impact of such reporting, as we have described, clearly demonstrates that it deserves closer attention. Next steps would engaging journalists involved in the reporting to determine what strategies clinical trialists, participants and stakeholders could utilize to improve reporting. An analysis of the strategies used by stakeholders to provide information to the media and the relative successes of these strategies would also be worth studying.

## Conclusion

In conclusion, our review identifies the heterogeneous reporting of issues related to these trial closures and the relatively poor involvement of stakeholders for the purpose of interviews. In several instances these concerns were not supported by evidence and were possibly inaccurate. However, the overall issues reported in the media reports is a sense of distrust and concern for the well-being of participants. Given the role of the media in reporting on the conduct of future prevention trials, serious initiatives are warranted to engage the media and preempt difficulties in the transference of information.

## Competing interests

The author(s) declare that they have no competing interests.

## Authors' contributions

EM, SS, EW conceptualized the study. EM, EW, SS, PW and BR carried out the searches and data abstraction. EM, EW, BR, PW, SS, and KW wrote the drafts of the manuscript. EM, EW, BR, PW, SS, and KW dealt with critical revisions and final submission of the manuscript. All authors approved the final manuscript.

## Pre-publication history

The pre-publication history for this paper can be accessed here:



## Supplementary Material

Additional File 1 Concerns cited in Cambodian reports. Click here for file

Additional File 2 Concerns cited in Cameroon reports. Click here for file

## References

[B1] (2004). UNAIDS: AIDS epidemic update. Geneva: UNAIDS.

[B2] Van Rompay KK, McChesney MB, Aguirre NL, Schmidt KA, Bischofberger N, Marthas ML (2001). Two low doses of tenofovir protect newborn macaques against oral simian immunodeficiency virus infection. J Infect Dis.

[B3] Van Rompay KK, Schmidt KA, Lawson JR, Singh R, Bischofberger N, Marthas ML (2002). Topical administration of low-dose tenofovir disoproxil fumarate to protect infant macaques against multiple oral exposures of low doses of simian immunodeficiency virus. J Infect Dis.

[B4] Subbarao S, Otten R, Ramos A, Jackson E, Adams D, Kim C, Bashirian S, Johnson J, Monsour M, Janssen R, Paxton L, Greenberg A, Folks T (2005). Chemoprophylaxis with oral tenofovir disoproxil fumarate (TDF) delays but does not prevent infection in Rhesus Macaques given repeated rectal challenges of SIV. 12th Conference on Retroviruses and Opportunistic Infections.

[B5] Cohen J (2004). AIDS research. Cambodian leader throws novel prevention trial into limbo. Science.

[B6] Cohen J (2005). Cameroon Suspends AIDS Study. Science, Science Now.

[B7] Cohen J (2005). AIDS clinical trials. More woes for novel HIV prevention approach. Science.

[B8] Chase M (2004). Key AIDS study in Cambodia now in jeopardy. The Wall Street Journal.

[B9] Singh JA, Mills EJ (2005). Abandoned trials of Pre-Exposure Prophylaxis for HIV: What went wrong?. PLOS Medicine.

[B10] IRIN-News (2005). CAMEROON: Clinical trial of anti-HIV drug on sex workers in question. http://www.irinnews.org/report.asp?ReportID=45252&SelectRegion=West_Africa.

[B11] Atatah C (2005). Douala AIDS Drug Controversy: Medical Council Says Trials Violated Ethical Norms. The Post News Line.

[B12] ACT-UP-Paris (2004). tenofovir: inethical trial. ACT-UP Paris.

[B13] Wilson K, Code C, Dornan C, Ahmad N, Hebert P, Graham I (2004). The reporting of theoretical health risks by the media: Canadian newspaper reporting of potential blood transmission of Creutzfeldt-Jakob disease. BMC Public Health.

[B14] Nerlich B, Clarke DD (2003). Anatomy of a media event: how arguments clashed in the 2001 human cloning debate. New Genet Soc.

[B15] James JS (2004). Cambodia stops important tenofovir prevention trial. AIDS Treatment News.

[B16] Munthit K (2004). Cambodian PM orders anti-AIDS drug test halted; cites human values.

[B17] Naik MCaG (2004). Key AIDS Study in Cambodia Now in Jeopardy. Wall Street Journal.

[B18] Ahmad K (2004). Trial of antiretroviral for HIV prevention on hold. The Lancet Infectious Diseases.

[B19] Stephan H (2004). Growing Pains in AIDS drug Development: HIV drug trial in Cambodia halted. Nature Biotechnology.

[B20] McKew M (2004). Drug Trial to start in Cambodia. The 7:30 report.

[B21] Russell S (2004). AIDS Chicago Antiviral Drug Used to Treat AIDS to be tested as Vaccine. San Francisco Chronicle.

[B22] JMO (2004). Pro-abortion Group Thwarted in attempt to Test Dubious HIV drugs on Cambodian Prostitutes. Phnom Penh: LifeSiteNews.

[B23] Alcorn K (2004). Cambodian study of tenofovir for HIV prevention halted, PM cites 'human rights'. AIDSMAP.

[B24] Timberg C (2004). Dose of Prevention where HIV thrives: Nigeria Brothel is test site for new pill.

[B25] Cohen J (2004). Cambodian Leader Throws Novel Prevention Trial into Limbo. Science.

[B26] Jeffreys E (2004). Cambodian Sex Workers win against Bill Gates. Scarlet Alliance.

[B27] Comments IN (2004). Cambodia's premier halts planned trials of AIDS drug. Life Issues Net.

[B28] Cairns G (2004). Sex Workers force end to drug trial. Positive Nation.

[B29] Canada.com (2004). Cambodia's prime minister halts AIDS drug trial. Science and Development Network.

[B30] Thomas J (2004). Cambodian HIV Clinical Trial impasse: UNAIDS mediation is essential. AIDS Asia.

[B31] Ojcius D (2004). HIV vaccine trial halted. Nat Rev Microbiol.

[B32] Munthit K (2004). PM wants anti-AIDS drug trials stopped. The Gazette.

[B33] Reenie S The Gilead Saga. American Journal of Bioethics Editors Blog.

[B34] Cannistra SA (2004). The ethics of early stopping rules: who is protecting whom?. J Clin Oncol.

[B35] Bernard EJ (2005). Cameroon study of tenofovir for HIV prevention suspended, Health Minister claims 'dysfunctions' in study protocol. Douala: Aidsmap.

[B36] AEGiS-IRIN (2005). CAMEROON: Clinical trial of anti-HIV drug on sex workers in question. Yaounde: Integrated Regional Information Network.

[B37] Colombant N (2005). Cameroon Activists Want Protection of Drug Test Victims. Abidjan: VOA.

[B38] Coalition GCfMatAVA (2005). Statement on the PREP trials in Cambodia and Cameroon. AF-AIDS.

[B39] publique J-LR-Mds (2004). Reply to posting by Act-UP Paris.

[B40] Samba-Kounzi R (2004). emed@usahealthgap.org.

[B41] Shetty P (2005). Cameroon suspends trial of AIDS drug after protests. Science and Development Network.

[B42] Tall DCT (2005). "Gilead Scandal in Cameroun" AfriCASO condemns. Actions Treatments.

[B43] AF-AIDS Tenofovir trials: Interview with Beth Robinson from FHI. HealthGap.

[B44] Chase M (2005). AIDS-Preventative Test Halted by Cameroon Amid Protests. The Wall Street Journal.

[B45] Simeon V (2005). SIDA: Le Scandale est dans l'eprouvette. La nouvelle Expression.

[B46] (2004). AIDS drug trial stopped in Cambodia. Washington Times.

[B47] (2004). Major HIV drug trial to be halted. BBC News.

[B48] J C (2004). The Asian Equation: One size doesn't fit all. International Antiviral Therapy Evaluation Center.

[B49] News B (2004). Major HIV drug trial to be halted. BBC News.

[B50] (2004). Cambodian sex workers reject HIV drug trials. Asian Labor News.

[B51] Ahmad K (2004). Trial of antiretroviral for HIV prevention on hold. Lancet Infect Dis.

[B52] Ethics, drug trials and HIV: the case of tenofovir.

[B53] James JS (2004). Cambodia stops important tenofovir prevention trial. AIDS Treat News.

[B54] Group NHVaMA (2004). Phase 2 trial of oral tenofovir use on a chemoprophylaxis for HIV infection in Nigeria: The outcome of community involvement with the scientific research process.

[B55] Sex workers trump Gates' humanitarian bid. Business Report.

[B56] Munthit K PM wants anti-AIDS drug trials stopped. The Gazette.

[B57] McCombs M, Shaw D (1972). The Agenda-Setting Function of the Mass Media. Public Opinion Quarterly.

[B58] Mills E, Cooper C, Guyatt G, Gilchrist A, Rachlis B, Sulway C, Wilson K (2004). Barriers to participating in an HIV vaccine trial: a systematic review. Aids.

[B59] Mills EJ, Singh S, Dolma S, Nixon S, Kapoor S (2005). Enrolling women into HIV prevention trials: A logistical challenge but ethical imperative. Presented at the 2005 Canadian HIV AIDS Research Conference.

[B60] Duan N (2005). Listening to consumers and HIV vaccine preparedness. Lancet.

[B61] Schuklenk U (2004). The standard of care debate: against the myth of an "international consensus opinion". J Med Ethics.

[B62] Lie RK, Emanuel E, Grady C, Wendler D (2004). The standard of care debate: the Declaration of Helsinki versus the international consensus opinion. J Med Ethics.

[B63] Steinbrook R (2000). Medical journals and medical reporting. N Engl J Med.

[B64] Lomas J (2000). Connecting Research and Policy. Canadian Journal of Policy Research.

[B65] Wilkie T (1996). Sources in science: who can we trust?. Lancet.

[B66] (2001). Social Issues Research Centre, Royal Society, Royal Institution of Great Britain. Guidelines on science and health communication. Oxford: Social Issues Research Centre. http://www.sirc.org/publik/revised_guidelines.shtml.

[B67] IAVI (2005). International AIDS Vaccine Initiative. http://www.iavi.org.

