# Supplementary material for: Media reporting of tenofovir trials in Cambodia and Cameroon
Source: BMC Int Health Hum Rights. 2005 Aug 24;5:6. doi: 10.1186/1472-698X-5-6 (PMC1242229; doi:10.1186/1472-698X-5-6)
Supplement: Additional File 1 — Concerns cited in Cambodian reports. [file 1472-698X-5-6-S1.doc]

# Additional file 1

# Concerns cited in Cambodian reports

|  | Alcorn ,Aug 13 2004 | Asian Labor News Mar 30 2004 | BBC News Aug 14 2004 | Business Report Feb 28 2005 | Timberg 2004 | Herrera Oct 1 2004 | Cohen Dec 2004 | Jeffreys Aug 16 2004 | Irving News Comments Aug 11 2004 | Positive Nation Nov 2004 | LifeSiteNews Aug 16 2004 | Science/ Dev Net Aug 13 2004 | Thomas Oct 5 2004 | Cohen Aug 20 2004 | James July 23 2004 | Munthit Aug 11 2004 | Chase Aug 12 2004 | Ahmad Oct 4 2004 | Russell Dec 1 2004 | UPI Aug 14 2004 | Obesity Fitness Wellness Week Aug 28 2004 | Ojcius, Oct 2 2004 | Munthit, Aug 12 2004 | Reenie, Feb 18 2004 |
| --- | --- | --- | --- | --- | --- | --- | --- | --- | --- | --- | --- | --- | --- | --- | --- | --- | --- | --- | --- | --- | --- | --- | --- | --- |
| **Who was interviewed?*** |  | I | I |  | P | O-1 | I,O-2 | R,A |  |  |  |  |  | A | A |  |  | O-3 |  |  |  |  |  |  |
| **Allegations** |  |  |  |  |  |  |  |  |  |  |  |  |  |  |  |  |  |  |  |  |  |  |  |  |
| Inadequate Counseling/Prevention Resources |  |  |  |  |  |  |  |  |  |  |  |  |  |  |  |  |  |  |  |  |  |  |  |  |
| Prevention limited to increase chance of infection |  |  |  |  |  |  |  |  |  |  |  |  |  |  |  |  |  |  |  |  |  |  |  |  |
| Too few support staff |  |  |  |  |  |  |  |  |  |  |  |  |  |  |  |  |  |  |  |  |  |  |  |  |
| Trial reduces incentive to practice safe sex |  |  |  |  |  |  |  |  |  |  |  |  |  |  |  |  |  |  |  |  |  |  |  |  |
| Exposes women to unnecessary chance of infection |  |  |  |  |  |  |  |  |  |  |  |  |  |  |  |  |  |  |  |  |  |  |  |  |
| Want medical insurance for participants |  |  |  |  |  |  |  |  |  |  |  |  |  |  |  |  |  |  |  |  |  |  |  |  |
| Who will be responsible for treatment after the trial? |  |  |  |  |  |  |  |  |  |  |  |  |  |  |  |  |  |  |  |  |  |  |  |  |
| Will not be able to afford drug, if effective |  |  |  |  |  |  |  |  |  |  |  |  |  |  |  |  |  |  |  |  |  |  |  |  |
| Desire lifetime medical care |  |  |  |  |  |  |  |  |  |  |  |  |  |  |  |  |  |  |  |  |  |  |  |  |
| Trial violates participants human rights |  |  |  |  |  |  |  |  |  |  |  |  |  |  |  |  |  |  |  |  |  |  |  |  |
| Researchers believe sex workers are a cheap resource |  |  |  |  |  |  |  |  |  |  |  |  |  |  |  |  |  |  |  |  |  |  |  |  |
| Suspicious of why research is being done in Cambodia |  |  |  |  |  |  |  |  |  |  |  |  |  |  |  |  |  |  |  |  |  |  |  |  |
| Exploitation of vulnerable population |  |  |  |  |  |  |  |  |  |  |  |  |  |  |  |  |  |  |  |  |  |  |  |  |
| Participants dislike experimentation |  |  |  |  |  |  |  |  |  |  |  |  |  |  |  |  |  |  |  |  |  |  |  |  |
| Study protocol unethical |  |  |  |  |  |  |  |  |  |  |  |  |  |  |  |  |  |  |  |  |  |  |  |  |
| Concern with drug safety |  |  |  |  |  |  |  |  |  |  |  |  |  |  |  |  |  |  |  |  |  |  |  |  |
| Long term health impacts |  |  |  |  |  |  |  |  |  |  |  |  |  |  |  |  |  |  |  |  |  |  |  |  |

I=Investigator; P=Participant; R=Representative; A=Activist; O=Others

- -1=NIAID representative, -2=Cambodia AIDS Authority, -3=Bioconsultant
