# Supplementary material for: Media reporting of tenofovir trials in Cambodia and Cameroon
Source: BMC Int Health Hum Rights. 2005 Aug 24;5:6. doi: 10.1186/1472-698X-5-6 (PMC1242229; doi:10.1186/1472-698X-5-6)
Supplement: Additional File 2 — Concerns cited in Cameroon reports. [file 1472-698X-5-6-S2.doc]

# Additional file 2

# Concerns cited in Cameroon reports

|  | Reenie  Feb 18 2005 | Chase  Feb 8 2005 | Tidiane Tall  Feb 15 2005 | Colombant Feb 15 2005 | Bernard Feb 18 2005 | ACT-UP Press Releases | AEGiS-IRIN  Jan 27 2005 | Shetty Feb 18 2005 | Af-AIDS Feb 17 2005 | Simeon 2005 | Rey  2004 | Samba-Kounzi  2005 | NHVMAG  2004 |
| --- | --- | --- | --- | --- | --- | --- | --- | --- | --- | --- | --- | --- | --- |
| Who was interviewed?* |  |  | A | I, A, |  | A | I,R,A | I | I |  | O | A | A |
| **Allegations:** |  |  |  |  |  |  |  |  |  |  |  |  |  |
| Inadequate counseling/prevention resources |  |  |  |  |  |  |  |  |  |  |  |  |  |
| Participants being told they were getting a definite preventive medicine |  |  |  |  |  |  |  |  |  |  |  |  |  |
| Consent forms/counseling only in English |  |  |  |  |  |  |  |  |  |  |  |  |  |
| Participants not fully informed of risks |  |  |  |  |  |  |  |  |  |  |  |  |  |
| Purposely being counseled to engage in high risk  behavior |  |  |  |  |  |  |  |  |  |  |  |  |  |
| Prevention package insufficient (ie. No female condoms) |  |  |  |  |  |  |  |  |  |  |  |  |  |
| Inadequate number of social workers/support staff/local help |  |  |  |  |  |  |  |  |  |  |  |  |  |
| No help for those who become infected during trial |  |  |  |  |  |  |  |  |  |  |  |  |  |
| Concerns for post-treatment care |  |  |  |  |  |  |  |  |  |  |  |  |  |
| Trial violates human rights |  |  |  |  |  |  |  |  |  |  |  |  |  |
| Participants used like guinea pigs/exploitation of vulnerable population |  |  |  |  |  |  |  |  |  |  |  |  |  |
| Study protocol unethical |  |  |  |  |  |  |  |  |  |  |  |  |  |
| Trial only to promote drugs commerical prospect/ financially cheaper to run in developing nation |  |  |  |  |  |  |  |  |  |  |  |  |  |
| Standards are different than developed country settings |  |  |  |  |  |  |  |  |  |  |  |  |  |
| Participant compensation is insufficient/ or unethical |  |  |  |  |  |  |  |  |  |  |  |  |  |

*I=Investigator; P=Participant; 3= Representative; A=Activist; O=Other (Local Physician)
